# The multi‐ethnic global lung initiative 2012 (GLI-2012) norms reflect contemporary adult's Algerian spirometry

**DOI:** 10.1371/journal.pone.0203023

**Published:** 2018-09-04

**Authors:** Abdelbassat Ketfi, Merzak Gharnaout, Mohamed Bougrida, Helmi Ben Saad

**Affiliations:** 1 Department of Pneumology, Phthisiology and Allergology, Rouiba Hospital, Algiers, University of Algiers, Faculty of Medicine, Algiers, Algeria; 2 Metabolic Diseases Research Laboratory, Faculty of Medicine, Constantine University, Constantine, Algeria; 3 Department of Clinical Physiology and Functional Explorations, BENBADIS Hospital, Constantine, Algeria; 4 Department of Physiology and Functional Explorations, Farhat HACHED Hospital, Sousse, Tunisia; 5 Laboratory of Physiology, Faculty of Medicine of Sousse, University of Sousse, Sousse, Tunisia; 6 Heart Failure Research Laboratory (LR12SP09), Farhat HACHED Hospital, Sousse, Tunisia; Massachusetts General Hospital, UNITED STATES

## Abstract

**Background:**

The validation of the multi-ethnic GLI-2012 spirometric norms has been debated in several countries. However, its applicability in Algeria has not been verified.

**Aim:**

To ascertain how well the GLI-2012 norms fit contemporary adult Algerian spirometric data.

**Methods:**

This was a cross-sectional study of a convenience sample of 300 healthy non-smoker adults (50% men, age range: 18–85 years) recruited from the Algiers region general population. All participants underwent a clinical examination and a plethysmography measurement. Z-scores for some spirometric data [FEV_1_, FVC, FEV_1_/FVC and forced expiratory flow at 25–75% of FVC (FEF_25-75%_)] were calculated. If the average Z-score deviated by “< ± 0.5” from the overall mean, the GLI-2012 norms would be considered as reflective of contemporary Algerian spirometry.

**Results:**

The means±SDs of age, height, weight, FVC, FEV_1_, FEV_1_/FVC and FEF_25-75%_ of the participants were, respectively, 48±17 years, 1.65±0.10 m, 73±14 kg, 4.04±1.04 L, 3.18±0.82 L, 0.79±0.05 and 4.09±1.09 L/s. Almost the quarter of participants were obese. The total sample means±SDs Z-scores were 0.22±0.87 for FVC, 0.04±0.88 for FEV_1_, -0.34±0.67 for FEV_1_/FVC and 0.93±0.79 for FEF_25-75%_. For men and women, only the means±SDs of the FEF_25-75%_ Z-scores exceeded the threshold of “± 0.5”, respectively, 1.13±0.77 and 0.73±0.76.

**Conclusion:**

Results of the present study, performed in an Algerian population of healthy non-smoking adults, supported the applicability of the GLI-2012 norms to interpret FEV_1_, FVC and FEV_1_/FVC but not the FEF_25-75%_.

## Introduction

Lung function tests are useful tools for diagnosing and monitoring a variety of adults’ chronic respiratory diseases **[1–3]**. Their outcomes are habitually reported as percentage predicted where predicted data are acquired from a healthy non-smoker norm population **[4–6]**. Nevertheless, predicted normal data from diverse sources may change widely, and as the variability of tests fluctuates with “time of life”, the use of percentage predicted leads to an age bias **[7–9]**. The age bias can be avoided by the use of sex, age, height and ethnicity specific Z-score **[10]**. The latter indicates how many standard-deviations (SDs) a measurement is from its predicted value, with only 5% of healthy subjects having a Z-score of 1.6445 or less (5^th^ percentile) **[10]**. Unlike percentage predicted, Z-score is exempt from bias due to age, height, sex and ethnic group, and is consequently useful in defining the lower and upper limits of normal ranges; they also simplify uniform interpretation of test records **[10]**.

According to scholarly societies (*eg*; American thoracic and European respiratory societies (ATS/ERS) **[4]**) “Ideally, norms are calculated with equations derived from measurements observed in a representative sample of healthy subjects in a general population” **[4]**. So, it is imperative to use norms that fit the population to be explored **[4]**. In Algeria, spirometric norms have been developed for adults living in Constantine, an Eastern region of Algeria, being 649 m above sea level (study population: 19–73 years) **[11]**. Nevertheless, up to now norms from the European Coal and Steel Community (study population: 18–70 years) **[12]** are extensively used despite having been found to cause misinterpretation of spirometric data in a significant proportion of North-African population (*eg*; Tunisian **[13]** and Algerian **[11]** ones). In 2012, the global lung initiative (GLI-2012) released spirometric norms derived from data collected from 72031 healthy individuals aged 3–95 years **[10]**. The spirometric values of 273 Algerian adults **[11]** were included in the Caucasian group (n = 55428). A major breakthrough was the application of a novel statistical technique (GAMLSS; www.lungfunction.org/files/GAMLSS-in-action.zip; last visit: 7^th^ August 2018) **[10]**. In Algeria, these multi-ethnic global all-age norms are now implemented by manufacturers of spirometric devices and will replace the applied local spirometric norms **[11, 12]**.

The fit of the GLI-2012 norms has been tested in some Caucasian populations, and reported results have been disagreeing **[14–23]**. On the one hand, some authors opted for their use to interpret spirometry, for example in the Australasian **[16]**, Norwegian **[20]**, German **[18]** and French **[17]** populations. On the other hand, the GLI-2012 norms seem unsuitable for clinical use in the Swedish **[14]**, Finnish **[19]** and Brazilian **[22]** populations. Moreover, a Chinese study **[24]**, concluded that GLI-2012 norms **[10]** showed unfavorable generalizability to their sample population. An African study **[23]** has demonstrated that Tanzanians aged 13–29 years, compared to the predicted values for Black populations inhabiting the GLI-2012 norms, scored relatively lower in some spirometric data with the exception of the FEV_1_/FVC (1^st^ s forced expiratory volume/forced vital capacity) ratio. A Nigerian study **[21]** confirmed the above conclusion and reported disparities between values obtained from their norms and those for Afro Americans using the GLI-2012 norms **[10]**. In the Arab world, and at the best of the authors’ knowledge, only one study ascertained how well the GLI-2012 norms fit contemporary adult Tunisian spirometric data **[15]**. However, while only Tunisian “healthy” adults were included, the authors generalized their results and concluded that GLI-2012 norms don’t reflect contemporary adult’s North-African spirometry **[15]**. This “generalization” is questionable for at least two methodological reasons. The 1^st^ one was the low percentage of females (19.6%) included in their sample (n = 489), which could “biased” conclusions. The 2^nd^ reason concerns the “unusual” recruitment method of the “healthy” adults, consisting of a population that undergoes spirometry at an occupational medicine group. Therefore, before accepting the “generalization” of the Tunisian study conclusion **[15]**, it seems that verifying the applicability of the GLI-2012 **[10]** norms for the Algerian population is crucial for care activities and research, and is urgently needed.

It is of unlimited meaning that the population from which the norms are derived is representative of the population under study **[14]**. The age scattering and other anthropometric, ethnic, socioeconomic and environmental factors should be equivalent since such factors can mark lung function **[14]**. Furthermore, the methodology for performing spirometric tests (*eg*; protocol and equipment) must be stringent **[4, 25]**. The external validation of the GLI-2012 norms is recommended **[9, 10]** and further evaluations of applicability from other parts of the world (particularly the Arab one) are required in order to verify the appropriateness in these areas. Hitherto, there is no publication evaluating the applicability of the GLI-2012 norms for Algerian adults. Since the GLI-2012 norms **[10]** may be unsuitable for use in Algerian adults’ population, it is essential that physicians are made aware of the potential consequences of adopting these norms for clinical decision-making **[10]**.

The aim of this study was to evaluate if the GLI-2012 norms **[10]**, although endorsed by several respiratory societies, are applicable for an adult Arab population resident in Algeria.

## Population and methods

### Study design

A cross sectional study was performed in the Department of Pneumology, Phthisiology and Allergology at the Rouiba Hospital, Algiers (186 m above sea level), Algeria. The study was conducted in compliance with the 'Ethical principles for medical research involving Human subjects' of the Helsinki Declaration (available from: http://www.wma.net/en/30publications/30ethicsmanual/pdf/ethics_manual_arabic.pdf; last visit: 7^th^ August 2018). The study was approved (approval number: 0601/2014) by the Rouiba Hospital (Algiers) Medical Advice and Ethics Commission [president: Pr. Ferhat Zebboudj (zebboudjferhat@gmail.com)]. Written informed consent was obtained from all participants.

The present project comprised two parts. The first one (the aim of this study) was to verify the applicability of the GLI-2012 norms **[10]** for some spirometric data in a sample of Algerian adults’ healthy participants (GLI-2012 validation group). The second part aimed to generate plethysmographic norms for Algerian adults’ healthy population (plethysmographic norms group).

### Study population

The target population consisted of a group of healthy participants aged 18 years and more. They were selected by convenience sampling from the acquaintances of patients hospitalized at the Department of Pulmonology, Phthisiology and Allergology, during the visit-period for example.

Only healthy participants with technically acceptable and reproducible spirometry maneuvers were included. The presence of *(i)* acute or past chronic diseases of the respiratory system (*eg*, presence of physician-diagnosed respiratory disease (such as asthma, chronic bronchitis, chronic obstructive pulmonary disease, emphysema, or tuberculosis); hospitalization for lung or chest conditions), *(ii)* heart diseases which may influence the respiratory system (*eg*, heart failure, arrhythmia, unstable angina or myocardial infarction, uncontrolled blood hypertension), *(iii)* a cigarette smoking history of more than one pack-years, *(iv)* obesity levels 2 or 3, and *(v)* a higher level of sports practice (> 5 hours per week) were applied as non-inclusion criteria **[15, 26, 27]**.

The total population was divided into two groups: GLI-2012 validation group (n = 300, 50.0% men) and plethysmographic norms (n = 491, 50.3% men).

### Data collection procedures

Medical data were collected using a simplified and modified medical questionnaire derived from the ATS division lung diseases questionnaire **[28]**.

The decimal age (accuracy to 0.10 years) was calculated from the date of measurement and the date of birth **[29]**. Standing height and weight were measured. Depending on calculated body mass index (BMI, kg/m^2^), participants were classified as **[30]**: underweight (BMI < 18.5 kg/m^2^), normal weight (BMI between 18.5 and 24.9 kg/m^2^), overweight (BMI between 25.0 and 29.9 kg/m^2^) and obesity (BMI ≥ 30.0 kg/m^2^). Obesity was classified as level-1 (BMI between 30.0 and 34.9.0 kg/m^2^), level-2 (BMI between 35.0 and 39.9.0 kg/m^2^) and level-3 (BMI > 40.0 kg/m^2^).

Plethysmography was carried out in the sitting position, and a nose clip was applied. All tests were performed between 9.00 am and 3.00 pm by only one qualified person (AK in the authors’ list). Plethysmographic measurements were performed with a body plethysmograph (Body-box 5500, MediSoft, Belgium), carefully following the ATS/ERS recommendations **[31, 32]**. The spirometer was calibrated daily with a 3-L syringe. The plethysmographic technique and especially the FVC maneuver, were previously described **[15, 31–36]**. Briefly, at least three reproducible FVC measurements were obtained **[31]**. FVC and FEV_1_, the best two out of the three selected tests, did not differ by more than 0.150 L (if FVC ≥ 1 L) or 0.100 L (if FVC < 1 L). The highest FVC and FEV_1_ were computed, even though the two data did not come from the same flow-volume curve **[31]**. The following flow-volume curve data were measured and/or calculated: FEV_1_ (L), FVC (L), FEV_1_/FVC ratio (absolute value), forced expiratory flow at 25–75% of FVC (FEF_25-75%_, L/s) and Z-scores (without unit). Algorithms and stand-alone software for the GLI-2012 norms **[10]** are freely available from *www*.*lungfunction*.*org* (last visit: 7^th^ August 2018). For the GLI-2012 **[10]**, software calculated Z-scores for FEV_1_, FVC, FEV_1_/FVC and FEF_25-75%_, and exported the results to a “.csv file” for manipulation in a spreadsheet.

### Statistical analysis

The distribution of quantitative variables was normal and results were expressed by their means±SDs and 95% confidence interval (95%CI). The obesity status results were expressed as numbers (relative frequencies).

The chi-square test was used to compare percentages. The Student t-test was used to compare anthropometric and spirometric data of men and women.

Height-, age- and sex- specific Z-scores for spirometric data were calculated using the GLI-2012 norms **[10]**. If there was an offset between the GLI-2012 norms **[10]** and test population (measured spirometric data), the expected Z-scores of the test population would have a mean of “> zero” and a *SD* of “> one” and would therefore be considered as statistically significant **[10]**. As done in some studies **[10, 15, 16, 37]** and according to a consensus established by the *GLI* scientific advisory panel (*http*:*//www*.*lungfunction*.*org*; last visit: 7^th^ August 2018), a Z-score of “> ± 0.5” was arbitrarily considered to be clinically significant.

The associations between Z-scores and sex or anthropometric data (age, height, weight and BMI) were evaluated, respectively, by t-tests and Pearson’s product-moment correlation “r”. The “r” was considered as “high”, “good”, “fair” or “weak”, when it was, respectively, “> 0.70”, between “0.50 and 0.70”, between “0.30 and 0.50” or “≤ 0.30” **[38]**. If the GLI-2012 norms **[10]** are applicable, no such high or good relationships should exist **[14]**.

All mathematical computations and statistical procedures were performed using a statistical software (Statistica Kernel version 6; Stat Software. France). Significance was set at the 0.05 level.

## Results

Among the 608 explored adults, 491 (80.76%) were considered as healthy participants with normal spirometry (they form the “plethysmography norms group”). Among them, 300 adults (150 women) were randomly included in the GLI-2012 validation group.

**Fig 1:**
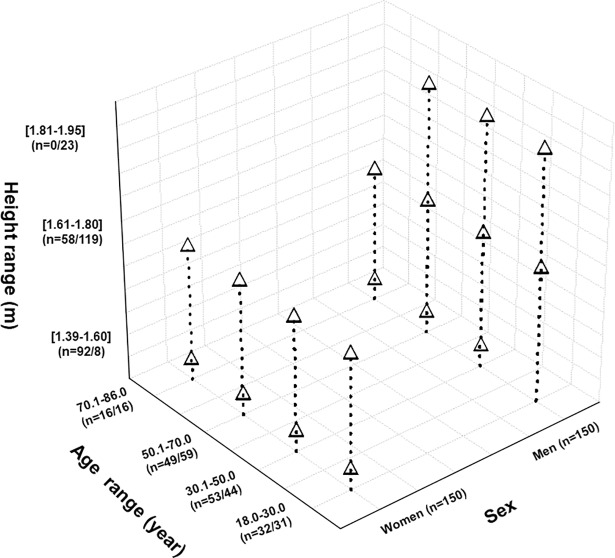
Distribution of the 300 participants according to sex, age and height ranges. **n: number.** Numbers between brackets (= X/Y) refer to the number of women (X) and men (Y).

**Fig 1** exposes the distribution of the GLI-2012 validation group according to sex, age and height ranges. The age distribution according to sex was similar; however, fewer participants aged > 70.1 years (10.7%) were included. No women were included in the height range 1.81–1.95 m and fewer men (5.3%) having a height range of 1.39–1.60 m were included.

**Table 1** exposes the anthropometric data of the GLI-2012 validation group. Women and men were age-, BMI- and obesity status- matched. Compared to women, men were significantly taller and heavier.

**Table 1:** Anthropometric data of the healthy non-smoker adults aged 18–85 years.

		Men (n = 150)	Women (n = 150)	Total sample (n = 300)
**Age (Yrs)**		48.53±17.38 (45.72 to 51.33)	46.77±17.15 (44.00 to 49.53)	47.65±17.26 (45.68 to 49.61)
**Height (m)**		1.72±0.08 (1.71 to 1.73)	1.58±0.07 (1.57 to 1.60)	1.65±0.10 (1.64 to 1.66)*
**Weight (kg)**		78±14.0 (76 to 81)	68±11 (66 to 70)	73±14 (71 to 75)*
**Body mass index (BMI, kg/m**^**2**^**)**		26.3±4.02 (25.7 to 27.0)	27.0±4.06 (26.3 to 27.6)	26.7±4.05 (26.2 to 27.1)
**Obesity status**	Underweight (BMI <18.5 kg/m^2^)	4 (2.66)	2 (1.33)	6 (2.00)
	Normal weight (BMI between 18.5 and 24.9 kg/m^2^)	54 (36)	49 (32.66)	103 (34.33)
	Overweight (BMI between 25.0and 29.9 kg/m^2^)	58 (38.66)	59 (39.33)	117 (39.00)
	Obesity level-1 (BMI between 30.0 and 34.9.0 kg/m^2^)	34 (22.66)	40 (26.66)	74 (24.66)

Data were mean±SD (95% confidence level), except for the obesity status, where data were number (%).

^*^p <0.05 (Student test or Chi-square test): men vs. women.

**Table 2** exposes the absolute values and Z-scores of some flow-volume curve data. Its main conclusion was that only FEF_25-75%_ was out of the range considered to be significantly different (< ± 0.5).

**Table 2:** Spirometric data of the healthy non-smoker adults aged 18–85 years.

		Men (n = 150)	Women (n = 150)	Total sample (n = 300)
**FVC**	L	4.72±0.92 (4.57 to 4.87)	3.36±0.63 (3.26 to 3.46)	4.04±1.04 (3.92 to 4.16)*
	Z-score	0.22±0.91 (0.07 to 0.37)	0.23±0.82 (0.09 to 0.36)	0.22±0.87 (0.12 to 0.32)
**FEV**_**1**_	L	3.70±0.73 (3.58 to 3.82)	2.67±0.54 (2.58 to 2.76)	3.18±0.82 (3.09 to 3.28)*
	Z-score	0.07±0.91 (-0.08 to 0.22)	0.00±0.85 (-0.14 to 0.14)	0.04±0.88 (-0.06 to 0.14)
**FEV**_**1**_**/FVC**	Absolute value	0.78±0.05 (0.78 to 0.79)	0.79±0.05 (0.79 to 0.80)	0.79±0.05 (0.78 to 0.79)
	Z-score	-0.26±0.66 (-0.37 to -0.15)	-0.41±0.68 (-0.52 to -0.30)	-0.34±0.67 (-0.41 to -0.26)*
**FEF**_**25-75%**_	L/s	4.78±0.98 (4.62 to 4.93)	3.41±0.70 (3.29 to 3.52)	4.09±1.09 (3.97 to 4.21)*
	Z-score	1.13±0.77 (1.00 to 1.25)	0.73±0.76 (0.61 to 0.85)	0.93±0.79 (0.84 to 1.02)*

**FVC:** forced vital capacity. **FEF**_**25-75%**_: forced expiratory flow at 25–75% of FVC. **FEV**_**1**_: 1^st^ s forced expiratory volume. Data were mean±SD (95% confidence level).

^*^p <0.05 (Student test): men vs. women.

**Table 3** exposes the “r” between spirometric Z-scores and anthropometric data. “Fair” correlations were found between age and FEV_1_ (total sample and women), between age and FEV_1_/FVC (total sample and men) and between height and FEF_25-75%_ (men and women). “Good” correlations were found only between age and FEF_25-75%_ (total sample, men and women). The spirometry Z-scores were not related to sex, except for FEF_25-75%_ and FEV_1_/FVC (**Table 2**).

**Table 3:** Correlation coefficient (r) between the spirometric Z-scores and the anthropometric data.

		FVC	FEV_1_	FEV_1_/FVC	FEF_25-75%_
**Total sample (n = 300)**	Age (Yr)	0.15*	0.31*	0.32*	0.60*
	Height (m)	-0.14*	-0.17*	-0.07	-0.09
	Weight (kg)	-0.12*	-0.15*	-0.06	-0.01
	BMI (kg/m^2^)	-0.03	-0.04	-0.01	0.07
**Men (n = 150)**	Age (Yr)	0.12	0.28*	0.33*	0.59*
	Height (m)	-0.18*	-0.28*	-0.23*	-0.36*
	Weight (kg)	-0.20*	-0.26*	-0.13	-0.18*
	BMI (kg/m^2^)	-0.14	-0.15	-0.02	-0.01
**Women (n = 150)**	Age (Yr)	0.18*	0.35*	0.30*	0.63*
	Height (m)	-0.20*	-0.27*	-0.15	-0.40*
	Weight (kg)	-0.03	-0.07	-0.10	-0.05
	BMI (kg/m^2^)	0.08	0.09	0.01	-0.20*

**BMI:** body mass index. **FEV**_**1**_: 1^st^ s forced expiratory volume. **FVC:** forced vital capacity. **FEF**_**25-75%**_: forced expiratory flow at 25–75% of FVC.

*Probability < 0.05.

## Discussion

The results of this study, performed in an Algerian population of 300 healthy non-smoking adults, supported the use of the GLI-2012 norms to interpret FEV_1_, FVC and FEV_1_/FVC but not the FEF_25-75%_.

To the best of the authors’ knowledge, only few studies **[14–22, 24]** aimed at evaluating the applicability of the GLI-2012 norms in healthy adults’ populations. **Table 4** presents the main characteristics and results of some studies reporting Z-scores data **[14–20]**.

**Table 4:** Main characteristics and results of some similar studies including “healthy” “non-smoker” adults.

1^st^ author	Sex	Country [Race]	Sample size	Age (Yrs)	% Men	Z-scores					Should GLI-2012 norms be used?
						FEV_1_	FVC	FEV_1_/FVC	FEF_25-75%_	Correlation with anthropometric data	
Hall et al. **[16]**	**TS**	Australia and New Zealand [Caucasian]	2066	40–84^**a**^	55.0	0.23±1.00	0.23±1.00	-0.03±0.87	0.07±0.95	.Weak associations with age, height and sex. .Associations of no physiological importance.	**YES** for the use of the GLI-2012 norms to interpret spirometry.
Ben Saad et al. **[15]**	**TS**	Tunisia [Arab]	489	18–60^**a**^ 37±9^**b**^ 23–53^**c**^	80.4	-0.55±0.87	-0.62±0.86	0.10±0.73.	NA	.Weak associations with age or height. .No association with sex.	**NO** for the use of the GLI-2012 norms to interpret spirometry
Backman et al. [14]	**TS**	Sweden [Caucasian]	501	22–91^**a**^	51.0	0.21±0.91	0.35±0.92	-0.25±0.85	NA	.Small associations with age, height, weight and sex.	**NO**. Compared to the ECSC norms, the GLI-2012 ones are superior, but not perfect.
Langhammer et al.[20]	M	Norway [Caucasian]	1035	20–90^**a**^ 52±15^**b**^	42.6	0.08±0.92	0.12±0.87	-0.09±0.82	NA	.No relevant correlation with age and height.	**YES.** The GLI-2012 norms are recommended for use.
	W		1403	20–90^**a**^ 56±16^**b**^		0.17±0.98	0.25±0.917	-0.20±0.78	NA		
Hüls et al. **[18]**	TS	Germany [Caucasian]	299 (at follow-up)	52–83^**a**^ 54±0.8^**b**^	0.0	-0.11±0.90	0.07±0.81	-0.35±0.79	NA	NR	**YES**. GLI-2012 can be used in longitudinal association analyses.
Hulo et al. **[17]**	M	France [Caucasian]	904	40–65^**a**^ 53±7^**b**^	45.9	0.01±1.11	0.18±1.00	-0.32±0.87	NA	NR	**YES.** The GLI-2012 norms can be used.
	W	Finland [Caucasian]	1067	40–65^**a**^ 53±7^**b**^		0.03±1.00	0.24±1.00	-0.40± 0.80	NA	NR	**NO.** The GLI-2012 predictions seem unsuitable for clinical use.
Kainu et al. **[19]**	M		387	19–82^**a**^ 50±16^**b**^	38.7	NR	0.37±1.00	-0.23±0.80	NA		
	W		613	18–83^**a**^ 48±16^**b**^		NR	NR	NR	NR		
	TS		1000	18–83^**a**^		0.25±1.04	0.37±1.00	NR	NR		

**ECSC:** European community for steel and coal. **FEF**_**25-75%**_: forced expiratory flow at 25–75% of FVC. **FEV**_**1**_: 1^st^ s forced expiratory volume.

**FVC:** forced vital capacity. **M:** men. **NA:** not applicable or not available. **NR:** not reported. **TS:** total sample. **W:** women.

Data were

^a^Minimum-maximum

^b^Mean±SD

^c^95% confidence interval.

### Methodology discussion

One of the main strong points of this study, as done in scarce relative ones **[14, 15]**, is its prospective design. Most of the remaining similar studies were retrospectives **[16–20]**. For example, all spirometric tests included in the 2012-Australian study **[16]** were performed in the year 2000 or later. Moreover, data included in the 2016-Norway **[20]**, in the 2016-German **[18]** and in the 2015-French **[17]** studies were derived, respectively, from four studies published between 2007 and 2014, from five studies published between 2005 and 2015 and from a study published in 2015. However, it was better to include more than one center, as done in some studies (n = 3 populations bases studies **[20]**, n = 4 locations **[19]**; n = 14 centers **[16]**).

According to the GLI group **[39]**, at least 150 men and 150 women are required to validate norms and to avoid spurious variances due to sampling mistake. The above criterion was applied in the similar studies, except in the Tunisian one **[15]**, where only 96 women were included **[Table 4]**. In order to avoid biased assessment of outcomes **[40]** and the sex-related effect on lung function **[41]**, similar percentages of men and women were included in this study. This was not the case of some others **[15, 18, 19, 23]**. For example, while the German study **[18]** included only women and the Tanzanian one only men **[23]**, in the Finnish **[19]** and the Tunisian **[15]** studies, women represented respectively, 61.3% and 19.6% of the total sample **(Table 4)**. In line with similar studies **[14, 15, 19, 20] (Table 4)**, the present one included adults with large age range (18 to 85 years, mean age: 48 years), a point that increases its external validity. Other related studies included either elderly adults aged 52 years and more **[18]** or adults with a narrow age range **[17]** or a mixture of children and adults **[23]**. Similar to some relative studies **[14–16, 18]**, only healthy never-smokers were included. This wasn’t the case for the Finnish study **[19]** where a history of less than 10 pack-years of smoking was allowed. Moreover, in the French study **[17]**, and in order to apply the GLI-2012 norms to “real-life” conditions in a general population, the authors have not taken into account smoking status. While, they noted that in a subgroup of non-smokers (n = 1081), the study sensitivity showed same results **[17]**, their approach is questionable. In the present study, the percentage of participant with an obesity level-1 was almost 25%. On the one hand, this was similar to the percentage reported in the Tunisian study **[15]**, where 20.3% of “healthy” participants were obese, and in line with the Finnish study **[19]**, where some participants with levels -1 and -2 were included (percentages not reported). On the other hand, 30% of the Algerian adults showed obesity **[42]**, and the present study group composition reflected this ‘‘healthy” population as they exist in the real population. This increases the external validity of the present study.

Similar to some studies **[14, 15, 17]**, only one type of spirometer was used, which ensures more intern validity for the reported data. In other relative studies, the use of several **[16]** or different **[20]** devices could be considered as a study limitation. As recommended, and as done in some studies **[14, 15, 17]**, the 2005-ATS/ERS guidelines for spirometry **[31, 32]** were applied. In some studies **[16, 18–20]**, all spirometry data were reported to be acceptable and repeatable as per the international spirometry guidelines relevant at the time of data collection (*eg*; 1994-ATS **[43]**).

The same statistical type of analysis applied in some relative studies **[14–16, 23]** was applied. However, the suggested fairly high cut-point of “0.5” for a significant mean difference to the GLI-2012 norms (equates to a difference of ~6% predicted **[15, 16]**) needs to be further appraised for its relevance in clinical medicine as well as in epidemiological studies. However, other statistical methods were applied. For example, Langhammer et al. **[20]** have advanced the following hypothesis: “if the GLI-2012 norms are appropriate, mean±SD Z-scores should approximate 0±1 across the entire age and height range studied”. In the Swedish study **[14]**, the agreement between the observed data in the local population and the GLI-2012 norms was verified and it was judged “perfect” if the mean Z-scores was zero and the SD was one. Moreover, relationships between Z-scores and age, height, weight and sex were examined and the lack of any such relationship was in favor of the GLI-2012 norms application **[14]**. In the Finnish study **[19]**, the difference between predicted FVC and FEV_1_ from their study and from GLI-2012 norms **[10]** was plotted as described by Bland and Altman **[44]**. In addition, the GLI-2012 norms were derived from cross-sectional data and application on longitudinal data, as done by Huls et al. **[18]** is encouraged at least for two reasons. Firstly, this could offer an original option to make longitudinal change of lung function comparable between different age groups and thereby substantially improve epidemiological analysis for respiratory risk factors **[18]**. Second, the use of norms makes it possible to appraise whether the change of lung function deviates from the its normal age-related decline **[18]**.

This study presented two limitations. The 1^st^ one concerned the non-determination of the participants’ socioeconomic levels and/or occupational status. As observed in the Tunisian study **[15]**, this could slightly influence the results, since there are significant differences in some spirometric data (*eg*, FEV_1_/FVC) depending on the general socioeconomic status **[45]**. The 2^nd^ limitation concerned the non-exclusion of participants with “possible” restrictive ventilatory defect or “lung hyperinflation”, as previously done in one study **[15]**. The main advanced reason to such choice was that the available local norms for lung volumes was published for the Eastern region of Algeria (Constantine, 649 m above sea level) and “seem” to be unsuitable for the Algiers region (186 m above sea level) **[11]**.

### Results discussion

The precision with which spirometry data are interpreted hinges on the suitability of the selected norms **[4, 37]**. Mistakes in interpretation, with respect both to overestimation and underestimation of lung function abnormalities, can arise if inappropriate norms are applied **[4, 37]**.

#### How well did the GLI-2012 norms fit contemporary Algerian spirometric data?

The ERS-GLI task force noted that data from some regions (*eg*; the Arab World) are urgently required **[10]**. This study results demonstrate that the GLI-2012 norms **[10]** are “well” matched to some spirometry outcomes obtained in a contemporary Algerian population using modern equipment and in accordance with international spirometry guidelines **[31]**.

As found in some studies **[16–18, 20] (Table 4)**, where means Z-scores for all measured spirometric data were “< ± 0.5”, in this study, FEV_1_, FVC and FEV_1_/FVC means Z-scores were less than the within test variation accepted in spirometry testing. Furthermore, the observed variability (SD of the Z-score) of the above outcomes **(Table 2)** was close to one, indicating a good overall fit. However, the above conclusion cannot be applied for the FEF_25-75%_ since its mean Z-score was “> 0.5” **(Table 2)**. This result was in opposition with the unique study **[16]** reporting FEF_25-75%_ data where its mean Z-score was 0.07±0.95 **(Table 4)**.

Z-scores point out how many SDs a measurement is from its normal value **[10]**. Compared to the percent predicted, they reduce bias due to age, height, sex and ethnic group, and are thus mainly helpful in defining the lower and upper limits of normal; they also simplify uniform interpretation of spirometry results **[15]**. In this study, there were good significant associations only between age and FEF_25-75%_ **(Table 3)** and only FEF_25-75%_ and FEV_1_/FVC Z-scores were related to sex (**Table 2**). These results support the use of the GLI-2012 norms to interpret FEV_1_, FVC and FEV_1_/FVC data in the Algerian population. Other authors tested this kind of association and found controversial results **[14–20] (Table 4)**. While some authors found some weak, but statistically significant, associations between the spirometry Z-scores and age **[14–16]**, height **[14–16]**, weight **[14]**, or sex **[14, 16]**, others didn’t find any association between the spirometry Z-scores and age **[20]** or height **[20]** or sex **[15]**. In the mutually adjusted multivariable models for some anthropometric data (*ie*; height, weight, age and sex), Thompson et al. **[37]** noted statistically significant but small associations for each of the spirometry Z-score results (FEV_1_ Z-scores declined with height and were lower in women, FVC Z-scores declined with height, FEV_1_/FVC Z-scores increased with age and were lower in women). According to some authors **[16, 37]**, the magnitude of any differences related to such associations was small and of no physiological importance. Two possible mechanisms for the observed relationship were advanced **[37]**: increased variability of spirometry data with age **[46]** or that the all-age norms **[46]** didn’t have sufficient data in the participants aged 60 years and more to accurately define the change in spirometry data with age.

#### Why did the GLI-2012 norms fit contemporary Algerian FEV_1_, FVC and FEV_1_/FVC data?

GLI-2012 datasets were obtained from 72 centers in 33 countries including Algeria **[10]**. Four ethnic groups were formed and the Algerian data **[11]** (n = 273) were included in the Caucasian group (n = 57395). Although representing almost 0.5% of the Caucasian data, the authors think that this inclusion could partially explain why the GLI-2012 norms fit contemporary Algerian spirometric data. Despite the ethnic, geographical, environmental, socio-economic status similarities between Tunisia and Algeria and despite very close anthropometric data between this study **(Table 1)** and the Tunisian one **[15] (Table 4)**, the two studies conclusions were opposite. One additional explanation, in addition to the above methodological differences, could be the existence of different subgroups in the North African population (Arab, Berber, Turkish descent) **[15]**.

#### Why didn’t the GLI-2012 norms fit contemporary Algerian FEF_25-75%_ data?

Two explanations could be advanced. The 1^st^ one is related to the high inter-test and intra-test variability of FEF_25-75%_ **[31]**. It is highly dependent on the validity of the FVC measurement and the level of expiratory effort **[31]**. For example, the FEF_25-75%_ between subject coefficient of variation varies between 20 and 62% **[10]**. This explains why it is not among the indices recommended by the ATS/ERS **[4]**. Moreover, the GLI group included it in their analyses only in response to requests from colleagues, especially those caring for children **[10]**. The 2^nd^ explanation is related to the effects of obesity on the FEF_25-75%_ **[43]**. On the one hand, almost 64% of the included participants were overweight or obese **(Table 1)**. On the other hand, it was shown that BMI was negatively associated with the FEF_25-75%_ **[47]** (*eg*, in this study the “r” between the BMI and the FEF_25-75%_ was significant at -0.12).

## Recommendation

In order to simplify comparative studies between countries, to avoid mistakes due to age-related gaps in norms **[48]** and to simplify the conversion to norms for diverse ethnic groups, the authors acclaim implementation of the GLI-2012 spirometric norms in healthcare in Algeria. While, local spirometric data are available for children aged 6 to 16 years **[49]**, there is a need to evaluate the applicability of the GLI-2012 in that age range.

In conclusion, the results of the current study support the use of the GLI-2012 norms to interpret clinical and research results in contemporary Algerian adults.

## Supporting information

S1 File Spirometric data of the 300 Algerian adults. (XLS)

## Abbreviations

ATS: American thoracic society
BMI: Body mass index
ERS: European respiratory society
FEV_1_: 1^st^ s forced expiratory volume
FVC: Forced vital capacity
GLI: Global lung initiative
FEF_25-75%_: forced expiratory flow at 25–75% of FVC
SD: Standard deviation
95%CI: 95% confidence interval
